# Melatonin in Plants – Diversity of Levels and Multiplicity of Functions

**DOI:** 10.3389/fpls.2016.00198

**Published:** 2016-02-19

**Authors:** Rüdiger Hardeland

**Affiliations:** Metabolism Research, Johann-Friedrich-Blumenbach Institute for Zoology and Anthropology, University of GöttingenGöttingen, Germany

**Keywords:** antioxidant, auxin-like, circadian, photoprotection, seeds, senescence, stress

## Abstract

Melatonin has been detected in numerous plant species. A particularly surprising finding concerns the highly divergent levels of melatonin that vary between species, organs and environmental conditions, from a few pg/g to over 20 μg/g, reportedly up to 200 μg/g. Highest values have been determined in oily seeds and in plant organs exposed to high UV radiation. The divergency of melatonin concentrations is discussed under various functional aspects and focused on several open questions. This comprises differences in precursor availability, catabolism, the relative contribution of isoenzymes of the melatonin biosynthetic pathway, and differences in rate limitation by either serotonin *N*-acetyltransferase or *N*-acetylserotonin *O*-methyltransferase. Other differences are related to the remarkable pleiotropy of melatonin, which exhibits properties as a growth regulator and morphogenetic factor, actually debated in terms of auxin-like effects, and as a signaling molecule that modulates pathways of ethylene, abscisic, jasmonic and salicylic acids and is involved in stress tolerance, pathogen defense and delay of senescence. In the context of high light/UV intensities, elevated melatonin levels exceed those required for signaling via stress-related phytohormones and may comprise direct antioxidant and photoprotectant properties, perhaps with a contribution of its oxidatively formed metabolites, such as *N*^1^-acetyl-*N*^2^-formyl-5-methoxykynuramine and its secondary products. High melatonin levels in seeds may also serve antioxidative protection and have been shown to promote seed viability and germination capacity.

## Introduction

Melatonin, once discovered in vertebrates as a hormone of the pineal gland, is now known to be formed in bacteria and numerous taxa of eukaryotes including various algae of different phylogenetic position and plants (Hardeland and Fuhrberg, 1996; Hardeland et al., 2007b; Tan et al., 2014b; Hardeland, 2015), whereas the presence of melatonin in archaea is still uncertain (Tan et al., 2014b). In addition to melatonin, recently discovered isomers of this molecule have been shown to also exist in some plants (Tan et al., 2014b). However, their full spectrum of abundance and their functional roles are not yet sufficiently known.

The fact that melatonin is an ancient molecule present in numerous phylogenetically distant organisms raises questions concerning the identity or similarity of functions. Some of them might be suspected to reflect fundamental cell biological requirements already existent before or at the basis of eukaryotic evolution. On the other hand, the billions of years since then may have been sufficient to allow the acquisition of secondary, additional functions that may entirely differ between taxa. This can be assumed to be also the case within the viridiplantae and even the embryophyta. In fact, strong evidence exists for such a functional diversity.

After the first discovery of melatonin in a phototrophic organism, the dinoflagellate *Lingulodinium polyedrum* (syn. *Gonyaulax polyedra*; Poeggeler et al., 1989, 1991; Balzer and Hardeland, 1991) and, thereafter, in macroalgae (Fuhrberg et al., 1996) and plants (Dubbels et al., 1995; Hattori et al., 1995), investigators tried to identify classic functions of this compound known from vertebrates, such as transmission of the signal ‘darkness’ and regulation of seasonality and circadian rhythmicity. The idea of possibly having found an agent mediating information on the length of scotophase, as in mammalian seasonal breeders, led to hopes concerning a role in plant photoperiodism. However, the first precondition, namely, existence of a high-amplitude circadian melatonin rhythm with a nocturnal maximum, was not generally fulfilled. Such a rhythm was demonstrated in *L. polyedrum* (Poeggeler et al., 1991; Balzer et al., 1993) and in a few plants, in particular, a short-day ecotype of *Chenopodium rubrum* (Kolář et al., 1997), but not generally in macroalgae and plants. In *C. rubrum*, no flower induction by melatonin was observed (Kolář and Macháčková, 2005). Instead, flowering was partially suppressed when given late at night. A similar effect was obtained with a putative pharmacological agonist, CGP 52608, considered as a ligand of an assumed mammalian nuclear melatonin receptor (Kolář et al., 1999). However, this transcription factor is actually no longer regarded as a melatonin-binding protein (Slominski et al., 2014). Moreover, neither melatonin nor its bioactive metabolite 5-methoxytryptamine induced flowering in several lemnaceans and in the crassulacean *Kalanchoë tubiflora*, another short-day plant (Hardeland and Poeggeler, 2003; Hardeland et al., 2007b). Therefore, a role in photoperiodism of plants seems unlikely, although this cannot be ruled out for other, phylogenetically distant phototrophic organisms, such as dinoflagellates (Balzer and Hardeland, 1991; Hardeland et al., 1995).

In recent years, transcriptomic, proteomic and metabolomic studies conducted in different plants such as *Arabidopsis thaliana* (Weeda et al., 2014; Qian et al., 2015), *Malus hupehensis* (Wang et al., 2014a) and *Cynodon dactylon* (Shi et al., 2015b,f) have revealed a plethora of melatonin-induced changes in the expression of genes at mRNA and protein levels as well as in metabolite concentrations. A remarkable diversity of actions was observed in different fields of function, which indicates a pleiotropic, orchestrating role of melatonin reminiscent of that known from animals (cf. Hardeland et al., 2011).

Within plants, another strong hint for a diversity of functions can be deduced from the extreme species- and organ-specific differences in melatonin concentration, which range from almost or totally undetectable to levels of above 20 or 30 μg/g, as summarized elsewhere (Hardeland et al., 2007b; Arnao, 2014). Recently, melatonin contents of up to 200 μg/g were reported to exist in kernels of several Iranian *Pistacia vera* cultivars (Oladi et al., 2014). It seems highly unlikely that such extremely high amounts serve the same function as low concentrations of a few pg/g, which would be in a range of vertebrate levels and may be compatible with the role of a signaling molecule. In dry seeds, under conditions of strongly reduced metabolism and gene expression and in the absence of circadian rhythmicity, melatonin might have a specific function differing from those in tissues with active metabolism (Balzer and Hardeland, 1996).

Even if the particular situation of seeds is left apart, other considerable differences exist, partially between organs, sometimes in the course of developmental processes, but most importantly between species and local variants. Notably, substantial variations were observed between specimens of the same or related species from habitats that differ with regard to temperature and light/UV exposure (cf. discussion in Hardeland et al., 2007b). It will be an important objective in the research of plant melatonin to understand why the levels of this compound can vary so profoundly and what the functional consequences of these divergencies are.

## Identity or Differences in Plant Melatonin Metabolism?

In plants, the classic pathway of melatonin biosynthesis from tryptophan comprises four steps, decarboxylation by tryptophan decarboxylase (TDC), hydroxylation of the amine by tryptamine 5-hydroxylase (T5H) to serotonin, its *N*-acetylation by a serotonin *N*-acetyltransferase (SNAT), which catalyzes the same reaction as the nonhomologous aralkylamine *N*-acetyltransferase (AANAT) of vertebrates, and the final *O*-methylation to melatonin by *N*-acetylserotonin *O*-methyltransferase (ASMT, formerly known as hydroxymethyl *O*-methyltransferase, HIOMT). For summaries using the actual terminology see Tan et al. (2014b) and Hardeland (2015). Whether or not these enzymes are exclusively responsible for melatonin formation in plants may be not as certain as it appears at first glance.

The highly divergent melatonin levels of different plant species raise the question of whether this may be only caused by strongly deviating expression levels of the same biosynthetic enzymes. Alternately, this divergency may be explained by either (i) splice variants, (ii) homologous gene variants encoding enzymes that substantially differ with regard to substrate affinity and *V*_max_, (iii) deviating precursor availability, or (iv) the involvement of enzymes of only moderate homology. This latter possibility is not generally unlikely, since this has been also discussed for melatonin synthesis in extrapineal sites of vertebrates (Hardeland, 2008), despite the observation that AANAT and ASMT are responsible for melatonin formation in the pineal glands and various other organs in these animals. For instance, another *N*-acetyltransferase, NAT-1, was reported to contribute to melatonin synthesis in the mammalian skin (Slominski et al., 2005). In insects, several AANAT subforms exist, which strongly differ in their substrate specificity and are also involved in other functions such as exoskeleton sclerotization or neurotransmitter catabolism (Han et al., 2012; Barberà et al., 2013; Hiragaki et al., 2015). In the future, isoenzymes of melatonin biosynthesis should be more generally considered in the botanical area. Evidence for such isoenzymes has been recently summarized (Hardeland, 2015). In principle, these may be isoforms coded by the same or duplicated genes, but could also represent functionally deviating members belonging to the same protein family. In particular, a lower substrate specificity can be associated with side activities in the indoleamine metabolism, as will be illustrated below by the example of *O*-methylation of *N*-acetylserotonin in plants. On the other hand, relatively small genetic deviations, even point mutations, can profoundly change the substrate specificity within an enzyme family. Therefore, homologies in nucleotide or amino acid sequences should not be overinterpreted with regard to a possible involvement in melatonin formation, as recently illustrated for some examples from plants (Hardeland, 2015).

Alternate pathways may already exist for the formation of serotonin from tryptophan. Serotonin biosynthesis in plants is usually believed to be carried out by the sequential actions of TDC and T5H and, therefore, to differ from the route in animals and dinoflagellates, which consists of tryptophan hydroxylation by a tryptophan 5-hydroxylase (TPH) followed by decarboxylation by an aromatic amino acid decarboxylase of usually broad substrate specificity (Hardeland, 2015). However, an animal-like pathway may be also present in some plant species. Although TDC and T5H have been shown to be decisive for serotonin synthesis in both monocots and dicots, such as rice (*Oryza sativa*) (Byeon et al., 2014c) and apple (*Malus* × *domestica*) (Lei et al., 2013), the rice TDC was also reported to decarboxylate 5-hydroxytryptophan (Park et al., 2008). The *T5H*-deficient Sekiguchi rice cultivar produced elevated levels of 5-hydroxytryptophan (Park et al., 2012), a finding that demonstrates the existence of an additional tryptophan hydroxylase activity independent of T5H. However, this rice variant did not exhibit an increase in melatonin because the more strongly accumulating tryptamine efficiently competed with serotonin at the SNAT enzyme. Surprisingly, transcriptional silencing of *T5H* in rice led to higher melatonin levels (Park et al., 2013a), a finding that, again, underlines the possibility of an alternate route of serotonin formation. Nevertheless, in rice, the classic plant pathway of TDC and T5H is obviously of higher relevance, since *TDC* overexpression causes an increase in melatonin (Byeon et al., 2014c). This may not be necessarily the case in all other plant species. In *Hypericum perforatum*, the expression of a tryptophan 5-hydroxylase was reported, in addition to TDC (He et al., 2012). Moreover, with reference to the statement made above that even point mutations can profoundly change the substrate specificity of some enzymes, it should be noted that the replacement of serine 372 by a glycine in the *Papaver somniferum* tyrosine decarboxylase generates a substantial affinity to 5-hydroxytryptophan (Torrens-Spence et al., 2014). In rice, three TDC isoforms were detected, TDC-1, TDC-2, and TDC-3, which seem to differ in their contribution to melatonin formation (Byeon et al., 2014c). The multiplicity of subforms and the general mutation-sensitive variability of substrate specificity observed in aromatic amino acid/amine decarboxylases (Torrens-Spence et al., 2013) may allow evolutionary changes in the serotonin pathway. Therefore, it may well be possible that, in some plants, the animal-type of serotonin formation has become prevalent. In the future, this should be particularly tested in plants that produce extremely high levels of melatonin.

Overexpression of *TDC* in rice leads to increased melatonin levels (Byeon et al., 2014c), a finding that indicates a rate limitation by the availability of tryptamine in this species. However, a second bottleneck in melatonin formation seems to exist in a number of plants, as can be deduced from the observation that serotonin concentrations are often by one or more orders of magnitude higher than those of melatonin, not only in the transgenic (Byeon et al., 2014c) but also in wild-type rice (Park et al., 2013d), in *Datura metel* (Murch et al., 2009), *Punica granatum* and *Fragaria* × *ananassa* (Badria, 2002). In these cases, especially when levels of *N*-acetylserotonin do not substantially exceed melatonin, SNAT seems to catalyze a secondary rate-limiting step, a situation reminiscent of vertebrate melatonin synthesis. However, this is not generally the case. In *Echinacea purpurea*, concentrations of serotonin and melatonin were in the same range (Jones et al., 2007). Serotonin contents that only moderately exceed those of melatonin may not substantially change the metabolic throughput in this pathway and seem to be a matter of variability between sibling species, strains and, perhaps, environmental differences of biotopes. For instance, serotonin concentrations did not substantially differ from those of melatonin in *Vaccinium macrocarpon*, but were almost five times higher in *V. vitis-idaea* (Brown et al., 2012). Notably, in several cases with very high melatonin contents, serotonin was reported to only moderately exceed the methoxyindole, such as in beans of *Coffea canephora* and *C. arabica* (serotonin 10.5 and 12.5 μg/g; melatonin 5.8 and 6.8 μg/g, respectively: Ramakrishna et al., 2012), or even to remain strongly below, such as in ripening wine grapes (serotonin about or lower than 10 μg/g; melatonin between about 100 and 150 μg/g: Murch et al., 2010) and developing flowers of *H. perforatum* (serotonin maximally about 2 nmol/g; melatonin maximally 4000 nmol/g: Murch and Saxena, 2002). Interestingly, the serotonin peak preceded that of melatonin in the *Hypericum* flower buds, whereas serotonin was practically undetectable in an early stage of *Vitis* grapes, although melatonin had already reached the range indicated. These findings strongly suggest that, in these high-melatonin tissues, *N*-acetylation of serotonin is not rate limiting. It would be of interest to study this more systematically and to test the possibility that a major difference between low- and high-melatonin plants may consist in the limitation or non-limitation of melatonin formation by SNAT activity, which might contribute to understanding why melatonin contents of plants can be so exceptionally divergent over many orders of magnitude.

Whether or not the deduced divergencies of SNAT activity may be associated with differently regulated subforms or the involvement of nonhomologous serotonin-acetylating enzymes is unknown. Pertinent information is still restricted to a very few species and most molecular data are from rice (cf. Hardeland, 2015). Recently, a *SNAT* from *A. thaliana* had been cloned (Lee et al., 2014b) and another one from a gymnosperm, *Pinus taeda* (Park et al., 2014). SNAT from *Oryza sativa* displays homology to a cyanobacterial enzyme and is, in line with this information, plastidially located (Byeon et al., 2013, 2014b). However, its vertebrate paralog, AANAT, is of alphaproteobacterial origin and thought to be inherited via mitochondria (Tan et al., 2013). To date, there is no existing evidence for mitochondrial serotonin acetylation in plants by an AANAT-like enzyme, but, on the small basis of respective knowledge, this should not yet be ruled out.

The final step of melatonin formation is catalyzed by cytosolic members of plant *O*-methyltransferases, which display differences to the mammalian ASMT (Park et al., 2013c) and do not seem to be closely related to the vertebrate enzymes. Obviously, several enzymes from plants are capable of catalyzing the *O*-methylation of *N*-acetylserotonin. In rice, three isoforms, ASMT1, ASMT2, and ASMT3 were reported to be encoded by different genes (Park et al., 2013b). In addition to these enzymes, ASMT activity was shown to be present in *Arabidopsis* caffeic acid *O*-methyltransferase (COMT). This activity was not negligible, although the nominal substrate, caffeic acid, displayed a higher affinity than *N*-acetylserotonin (Byeon et al., 2014a). Recently, *O. sativa* COMT was shown to methylate *N*-acetylserotonin at a 609-fold rate compared to ASMT1 from this species. Moreover, it was possible to increase or decrease melatonin synthesis in rice by overexpressing or suppressing *OsCOMT* (Byeon et al., 2015a). Nevertheless, it remains to be clarified to which degree other, competing substrates may reduce the ASMT-like activity of physiological OsCOMT levels. The situation seems to be entirely different in *Arabidopsis*, in which the specificity balance of COMT was anyway strongly on the side of caffeic acid. In fact, a recent study reported cloning and characterization of an AtASMT devoid of COMT activity (Byeon et al., 2016). However, this enzyme also methylated efficiently serotonin to 5-methoxytryptamine (5-MT), a finding that indicates the possible existence of an inverse route of melatonin formation, in which serotonin is first *O*-methylated and 5-MT, which is also accepted by SNAT, subsequently *N*-acetylated. Both routes, the traditional and the inverse one, may be used in parallel, as had been previously shown in *Saccharomyces*, in which melatonin can be formed from either *N*-acetylserotonin or 5-MT (Sprenger et al., 1999). According to the recent publication on AtASMT, ASMT-like sequences had been also detected in various other monocot and dicot species, however, with relatively low homology. An *ASMT* from *M. zumi* was cloned, characterized and also expressed in *A. thaliana* (Zuo et al., 2014). Overexpression of the transgene caused two to fourfold rises of melatonin in *A. thaliana*. With regard to the divergent findings on ASMT activities, three main conclusions should be drawn. (1) There may be considerable differences between species concerning the *O*-methyltransferases involved. These deviations may contribute to the remarkable differences in the melatonin levels detected in the various species. (2) The possibility of further enhancing melatonin formation by overexpressing *ASMT* transgenes indicates that ASMT activity can become rate limiting under certain conditions. This should not be seen as an unusual exception, since this is also known from mammals, in which AANAT is limiting at low or moderately elevated rates of melatonin synthesis, whereas ASMT can become limiting at highest rates of melatonin formation (Liu and Borjigin, 2005). (3) The variable substrate affinity and sometimes low specificity of *O*-methyltransferases should be taken as a caveat for not precociously concluding from partial homology on functional identity.

In plants, the understanding of catabolic melatonin metabolism is still in its infancy. From a general point of view, enzymatic and nonenzymatic routes of catabolism have to be distinguished. Nonenzymatic reactions with free radicals or singlet oxygen should be similar as in animals, but may be even more important, with regard to higher rates of oxidant generation in plants and more intense and poorly protected light exposure.

**FIGURE 1:**
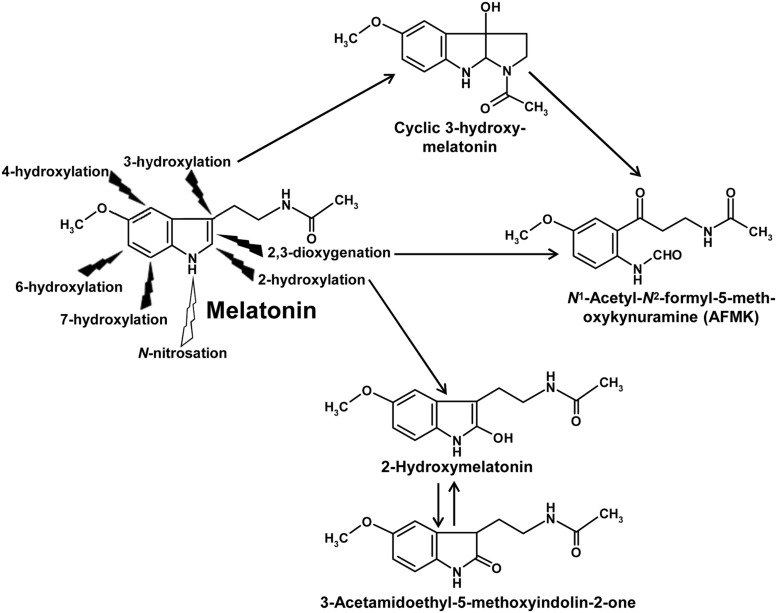
**Overview of ubiquitous primary metabolic reactions of melatonin.** All hydroxylation reactions are nonenzymatically possible by interaction with free radicals, e.g., by consecutive reactions with two ⋅OH. By contrast with animals, enzymatic 6-hydroxylation by CYP monooxygenases does not seem to play a substantial role in plants. Instead, 2-hydroxylation by M2H enzymes, which belong to the family of 2-oxoglutarate-dependent dioxygenases (2-ODDs), has been shown to be of quantitative importance. 2-Hydroxymelatonin is in a tautomeric equilibrium with 3-acetamidoethyl-5-methoxyindolin-2-one. 2,3-Dioxygenation is enzymatically possible, e.g., by peroxidases and other heme enzymes, as well as nonenzymatically by some photocatalysts, hemin, free radicals and singlet oxygen. AFMK is also formed from cyclic 3-hydroxymelatonin, e.g., by two ⋅OH. AFMK can be further metabolized to numerous other compounds not depicted because of lacking documentation in plants. *N*-nitrosation has been shown to occur by interaction with ⋅NO, other NO congeners or via transnitrosation from organic nitroso compounds. Additional reactions known from animals, such as deacetylation or demethylation, have not been sufficiently studied in plants.

An overview of nonenzymatic hydroxylation (Tan et al., 2002) and dioxygenation reactions has been included in a recent review article (Hardeland, 2015). Nonenzymatic hydroxylations in different positions of the indole moiety are caused by interactions with free radicals of higher reactivity, usually hydroxyl radicals (⋅OH), e.g., by sequential actions of either two ⋅OH or another hydrogen-abstracting radical followed by combination with ⋅OH. Among the hydroxylated products, a metabolite carrying a third ring should be especially mentioned, cyclic 3-hydroxymelatonin (**Figure 1**), which is formed nonenzymatically in animal tissues under conditions of oxidative stress and displays, reminiscent of the parent compound, properties of an antioxidant (Tan et al., 1998, 2014a). Nonenzymatic dioxygenation of melatonin is mostly caused by sequential actions of an electron/hydrogen-abstracting free radical and a superoxide anion (Hardeland et al., 2003) or by interaction with a singlet oxygen (de Almeida et al., 2003). During dioxygenation, the pyrrole ring is cleaved to give a substituted kynuramine, *N*^1^-acetyl-*N*^2^-formyl-5-methoxykynuramine (AFMK; **Figure 1**). Further possibilities including pseudoenzymatic and photocatalytic reactions that lead to the same metabolite have been summarized elsewhere (Hardeland et al., 2009). AFMK can be also formed from cyclic 3-hydroxymelatonin by interaction with two ⋅OH. Reactions of AFMK with free radicals can lead to the formation of numerous other metabolites (Rosen et al., 2006; Hardeland et al., 2009), which have, however, not yet been studied in plants.

With regard to enzymatic melatonin catabolism, several possibilities known from animals and other eukaryotes may exist, such as hydroxylations, demethylation, deacetylation, and dioxygenation. Because of the high numbers of cytochrome P_450_ (CYP) isoforms present in plants (Paquette et al., 2000; Zhong et al., 2002), hydroxylations and demethylation are highly likely. Among these possibilities, to date only one hydroxylation reaction has been detected, which was, however, not attributed to CYP enzyme, but rather to a melatonin 2-hydroxylase (M2H; Byeon et al., 2015b). M2H belongs to the 2-oxoglutarate-dependent dioxygenases (2-ODDs), which transfer the second O-atom to 2-oxoglutarate, which, thereafter, decomposes to succinate and CO_2_. Its gene from rice has been cloned and four different 2-ODD proteins were detected and shown to 2-hydroxylate melatonin (Byeon and Back, 2015). In plants, the M2H route seems to be of much higher importance than any other catabolic pathway of melatonin. In numerous plants such as rice, various dicots and also two gymnosperms, 2-hydroxymelatonin exceeded the melatonin levels, often by far (Byeon et al., 2015c). Notably, 2-hydroxymelatonin enters a tautomeric equilibrium with the relatively stable 3-acetamidoethyl-5-methoxyindolin-2-one (**Figure 1**), which can be easily detected in chromatograms containing the 2-hydroxylated metabolite (Hardeland, 2010). This indolinone is much more lipophilic than its tautomer, 2-hydroxymelatonin. Therefore, it might be of interest to find out whether the very high amounts of the latter compound may be related to the entrance of the indolinone into lipophilic compartments or into vacuoles in which a further metabolization is less likely, but from which 2-hydroxymelatonin may be tautomerically regenerated. The presence and functional capacity of M2H enzymes represents a major difference to melatonin catabolism in animals. With regard to the high levels of 2-hydroxymelatonin, it may be important to seek for eventual physiological roles of this compound or, alternately, to identify it as waste molecule.

Apart from this metabolite, only one hydroxylated product of melatonin was to date discovered in plants, namely 4-hydroxymelatonin, which made up not more than 0.05% of these metabolites, whereas 6-hydroxymelatonin, the major metabolite in animals, remained below the detection threshold (Byeon et al., 2015c). Dealkylation by CYP isoforms, which is a rather common reaction type of these enzymes, has not yet been documented in plants, perhaps, because researchers did not study this directly, and since the resulting product *N*-acetylserotonin would not have been distinguished from the same molecule as a precursor. Deacetylation of melatonin to 5-MT, another bioactive metabolite known from animals and also from phototrophic dinoflagellates (Hardeland et al., 1997, 2007b; Hardeland, 1999), has not yet been studied in plants. However, as mentioned above, 5-MT can be formed from serotonin by AtASMT (Byeon et al., 2016).

Enzymatic formation of the dioxygenated product, AFMK, has been shown to exist in rice, in which an indoleamine 2,3-dioxygenase (IDO) carrying out this reaction was identified and cloned. Upon overexpressing it as a transgene in tomatoes, melatonin levels were reported to decrease (Okazaki et al., 2010). This may indicate a certain physiological role of enzymatic AFMK formation, although the quantitative relevance cannot yet be easily judged. Quantification of AFMK in plant tissues was convincingly carried out in *Eichhornia crassipes* (Tan et al., 2007). Under natural light/dark conditions, the compound exhibited a 24-h rhythm with a maximum around the transition from photo- to scotophase, roughly in parallel with a rhythm of melatonin in this species. However, it is not yet clear to which extent this was due to an IDO and whether photochemical reactions, e.g., by singlet oxygen, contributed to the increase of AFMK over the photophase.

## Auxin-Like Effects

Morphogenetic and growth effects of melatonin, which comprise auxin-like actions, have been fully or partially reviewed a number of times in the last years (Arnao and Hernández-Ruiz, 2006, 2014, 2015; Paredes et al., 2009; Tan et al., 2012; Arnao, 2014; Hardeland, 2015). These observations have been made in various different species, both monocots and dicots, and different test systems, such as poacean coleoptiles (*Phalaris*, *Triticum*, *Avena*, *Hordeum)*, *Lupinus* hypocotyls, root growth and formation or regeneration of adventitious and lateral roots (*Oryza*, *Lupinus*, *Prunus*, *Arabidopsis*, *Brassica*), and shoot multiplication by nodal segments (*Mimosa*), as recently summarized (Hardeland, 2015). Notably, concentration differences for stimulatory or inhibitory effects in roots, as known from auxins, were also observed with melatonin.

Although, in phenomenological terms, the auxin-like effects are well documented, the mechanistic understanding of melatonin’s actions is still insufficient. The structural differences between the indolic compounds melatonin and indole 3-acetic acid (IAA) are too pronounced to assume affinity to the same binding sites. Indirect effects of melatonin on IAA levels have been reported, but did not reveal consistent changes. In *Brassica juncea*, 0.1 μM melatonin elevated the IAA concentration in roots (Chen et al., 2009). However, overexpression of an ovine *AANAT* transgene in ‘Microtom’ tomato plants caused an increase in melatonin, but a reduction in IAA that was associated with a loss of apical dominance (Wang et al., 2014b). Therefore, melatonin is not just a stimulator of IAA formation, but the actions of the two signal molecules can be dissected. However, the inverse correlation between melatonin and IAA observed in tomato may not necessarily reflect a physiological relationship, if the melatonin and IAA biosynthetic pathways compete for precursors and AANAT overexpression favors the former. However, this assumption would require direct experimental support. Another theoretically possible explanation might have been a catabolic route from melatonin via 5-MT and 5-methoxyindole 3-acetaldehyde to the auxin 5-methoxyindole 3-acetic acid, a pathway that exists in phototrophic dinoflagellates and animals (Hardeland et al., 2007b). However, this was ruled out in plants for reasons of product quantities (Arnao and Hernández-Ruiz, 2006). To date, it seems that the actions of melatonin and auxins are transmitted by different signal transduction pathways, which finally converge at some but not necessarily all regulatory checkpoints. The involvement of cytosolic calcium in both melatonin and IAA signaling, as recently discussed (Hardeland, 2015), would require further in-depth elaboration of mechanistic details. Differences in the actions of melatonin and auxins may be overlooked when studying a single or a very few growth-related or morphogenetic endpoints. In *A. thaliana*, melatonin as well as IAA and another auxin stimulated lateral root formation, but melatonin did not enhance the expression of an auxin-dependent GUS reporter (Koyama et al., 2013). An even more conflicting result was obtained in a transcriptome analysis of *A. thaliana*, according to which the genes of auxin signaling were preferentially downregulated by melatonin (Weeda et al., 2014). Under the impression of these findings, the auxin-like actions of melatonin appear more enigmatic than before. Another theoretical possibility is still devoid of direct experimental support and also seems to conflict with the transcriptome data. Melatonin was shown to downregulate IAA17 (indole-3-acetic acid inducible 17, alias auxin resistant 3, AXR3; Shi et al., 2015d), which acts as transcriptional repressor of various auxin-inducible genes. Therefore, melatonin might indirectly cause upregulations of auxin-dependent genes and, thus, cause effects known from IAA. However, these melatonin effects had been observed in the context of leaf senescence and may not be generally applicable.

Under the aspect of the highly divergent melatonin concentrations measured in plants, it should be underlined that relatively low concentrations of melatonin are required for auxin-like actions. This is precisely what one would expect from a signal molecule. The problem that arises is rather that of the role of melatonin in those species, their organs or seeds, which contain by orders of magnitude higher concentrations of melatonin. If, in these high-melatonin plants or parts thereof, melatonin were freely diffusible, regulation mechanisms based on high-affinity binding sites would no longer be functional, because of a persistent full saturation or even saturation-dependent desensitization. Therefore, the alternative seems to be that either melatonin is not freely diffusible, but sequestered by proteins, absorbed by oil droplets, perhaps otherwise hindered to leave certain compartments, or regulation mechanisms working at low concentrations are switched off. In the latter case, auxin-like effects of melatonin should not be expected.

The observation of growth inhibition at elevated levels of melatonin, especially in roots, has been forwarded as another argument for an auxin-like action. However, despite the similarity to the concentration dependence of auxin effects, this conclusion may not be entirely firm, because elevated levels of melatonin can interfere with the cytoskeleton including the mitotic spindle. This is known from the earliest studies of melatonin in plants, in endosperm cells of *Scadoxus multiflorus* (syn. *Haemanthus katherinae*; Jackson, 1969) and onion roots of *Allium cepa* (Banerjee and Margulis, 1973).

## Stress and Senescence

Melatonin effects in the complex of biotic and abiotic stress, defense, wound healing and senescence represent an emerging field, which seems to receive increasing attention. Notably, the transcriptome analysis by Weeda et al. (2014), which revealed down- rather than upregulations of auxin-related pathways in *A. thaliana*, demonstrated various stimulatory actions of melatonin on the pathways of ethylene, abscisic, jasmonic, and salicylic acids. However, in the context of stress, melatonin was also reported to conversely downregulate formation and upregulate catabolism of abscisic acid in *Malus* species (Li et al., 2015). In addition to the effects that seem to be mediated by these phytohormones, the role of melatonin as an antioxidant may play an additional role that could be in favor of stress resistance, healing and survival. In this regard, the situation is reminiscent of that in animals and comprises reduction of oxidant formation, direct scavenging of oxidants as well as induction of antioxidant enzymes and support of favorable redox balances of other antioxidants such as glutathione (Kostopoulou et al., 2015; Li et al., 2015; Shi et al., 2015f). This parallel to animals, which is not equally detectable in other functions of melatonin, might be seen as a consequence of a most ancient antioxidant role of melatonin (Hardeland et al., 1995), which is already demonstrable in unicellular organisms (Antolín et al., 1997) and includes mechanisms that reduce free-radical formation (Hardeland, 2005).

**FIGURE 2:**
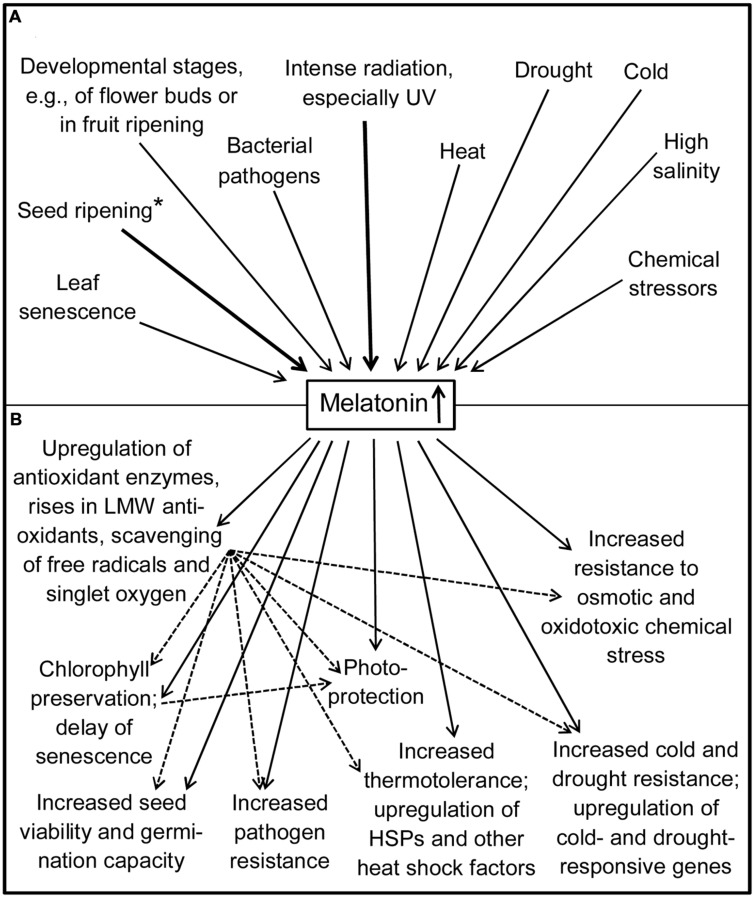
**Increases in melatonin (A) and their consequences for protection and stress tolerance (B).** Bold arrows: particularly strong accumulations of melatonin. Dotted arrows: secondary effects via antioxidative protection. ^∗^Melatonin may be more strongly taken up than synthesized on-site. HSP, heat shock protein; LMW, low-molecular weight. Some actions may be species-specific or conditional.

The complex of stress resistance, healing, and senescence has been fully or partially reviewed in recent publications (Arnao and Hernández-Ruiz, 2014, 2015; Hardeland, 2015; Zhang et al., 2015) or extensively addressed (Shi et al., 2015f). Instead of repeating in detail the findings summarized there, only their essence shall be briefly outlined, followed by a focus on the most recent results. A remarkable observation has been that the various forms of environmental or biotic influences that may be interpreted as stress typically lead to increases in melatonin (**Figure 2**). This includes extreme temperatures – both cold stress and heat stress –, intense radiation, drought, high salinity, and chemical stressors such as hydrogen peroxide, ZnSO_4_ or herbicides (for details see Arnao and Hernández-Ruiz, 2009b, 2013, 2014, 2015; Arnao, 2014; Hardeland, 2015; Zhang et al., 2015), and also exposure to a bacterial pathogen (Qian et al., 2015; Shi et al., 2015a). The rises in melatonin, caused by entirely different factors, may be indicative of its involvement in a fundamental stress resistance mechanism. Most of these observations had been made in various plant species, dicots and monocots. Increases of melatonin have been particularly described as a consequence of UV radiation, which will be discussed in the following section on photoprotection.

With regard to the complex of stress responses, as induced by various abiotic or biotic factors, of wound healing and leaf senescence, there is a considerable overlap of actions by several pertinent phytohormones, such as ethylene, abscisic, salicylic, and jasmonic acids/methyl jasmonate (Jibran et al., 2013; Khan et al., 2014), and also in their nexus with melatonin (Hardeland, 2015). This view is in line with the observation that melatonin supports the resistance to different forms of stress caused by factors as divergent as drought, cold, heat, osmotic stress, oxidative stress (Chan and Shi, 2015; Shi et al., 2015b) and also by bacterial pathogens (Shi et al., 2015a). Despite these similarities, the factors influenced by melatonin can be different according to the type of challenge. Nevertheless, joint pathways and response types do exist, especially in the cases of cold and drought stress. Moreover, it has to be noted that many forms of stress are associated with increased oxidative damage. Therefore, the antioxidant actions of melatonin appear to be a common theme of stress management in plants, a conclusion that has been similarly applied to many animals.

Among the most recent developments in this field of plant responses, a considerably increased number of studies has been conducted in *Arabidopsis*. With regard to an improved cold tolerance by melatonin, the overlap with drought resistance has become obvious by the induction of transcription factors involved in either of these responses, in particular, *CAMTA1*, *CBF1*, *CBF2a*, *CBF2b*, *CBF3a*, and *CBF3b* (Bajwa et al., 2014). Moreover, the cold-responsive genes *COR15a*, *RD22*, and *KIN1* (Bajwa et al., 2014; Shi et al., 2015c) as well as the oxidant-induced zinc-finger transcription factors *ZAT10* and *ZAT12* were upregulated by melatonin. The connection between cold tolerance and avoidance of oxidative damage was further confirmed and focused on another zinc-finger protein, ZAT6 (Shi and Chan, 2014). Knockdown of the *ZAT6* gene reduced the melatonin-mediated cold resistance, whereas *ZAT6* overexpression enhanced it. Additionally, melatonin was shown to upregulate *ZAT6* as well as *CBF1*, *CBF2*, and *CBF3*.

In the case of heat stress, other signaling pathways are, expectably, involved. In *Arabidopsis*, melatonin was recently shown to upregulate class A1 heat shock factors (HSFA1s). HSFA1-inducible genes such as *HSFA2s*, *HSA32* (heat stress-associated 32), *HSP90*, and *HSP101* are assumed to participate in the increased thermotolerance conveyed by melatonin (Shi et al., 2015e). With regard to other forms of stress, drought resistance was shown to be enhanced in *Arabidopsis* by overexpression of an ASMT transgene from *M. zumi* and, thus, by enhanced melatonin synthesis (Zuo et al., 2014). Another aspect of drought tolerance was investigated in *M. hupehensis* (Li et al., 2015). In this study, the antioxidant capacity of melatonin was demonstrated by decreases of H_2_O_2_ levels, in conjunction with upregulations of catalase and peroxidases. Some minor increases in ascorbate peroxidase (APX) were also reported, which were, however, more pronounced in *M. prunifolia*, a species with higher natural drought tolerance. Additionally, the protective effects of melatonin were associated with increased stomatal aperture and moderate improvements of photosynthesis. In *Glycine max*, melatonin enhanced both drought resistance and salt tolerance, in conjunction with a reversal of salt-induced reductions in the expression of numerous genes (Wei et al., 2015). Increased salt tolerance was also recently demonstrated in *O. sativa* (Liang et al., 2015) and in seedlings of *Citrus aurantium* (Kostopoulou et al., 2015). In the latter case, combinations of melatonin and ascorbic acid were tested, which increased the levels of several low molecular-weight antioxidants and activities of antioxidant enzymes. As in this study, many of the recent investigations revealed a causal relationship between exogenous stress factors and induction of oxidative stress and damage, which were mitigated by melatonin (cf. **Figure 2**). This was especially observed in dicots such as *Arabidopsis* (Shi and Chan, 2014) and *Malus* (Li et al., 2015), and monocots such as *Cynodon* (Shi et al., 2015b,f) and *Oryza* (Liang et al., 2015). For further details reviewed earlier see Tan et al. (2012). In *Cynodon*, melatonin was shown to reduce reactive oxygen species and lipid peroxidation (Shi et al., 2015b). In *Oryza*, an H_2_O_2_-overproducing mutant, *noe1*, was also tested, in which melatonin protected against accelerated leaf death (Liang et al., 2015). This finding may be also seen in the context of normal leaf senescence, since melatonin reduced chlorophyll degradation and suppressed senescence-associated genes. Protection by melatonin against losses of chlorophyll and delay of senescence appear to be a common theme in plants (for further details and mechanistic aspects see: Hardeland, 2015). Among the most recent investigations, the effects of melatonin on gene expression during leaf senescence, as revealed by a proteomic approach, should be mentioned (Wang et al., 2014a), and also a study in *Arabidopsis* on the relationship to *IAA17* (Shi et al., 2015d). While *IAA17* overexpression caused advanced senescence, melatonin downregulated this gene as well as the levels of *SEN4* (*senescence 4*) and *SAG12* (*senescence-associated gene 12*) mRNAs, thereby decelerating senescence.

The reduction of oxidative stress by melatonin has been recently investigated in the context of phytotoxic agents. In *Arabidopsis* treated with paraquat (=methyl viologen), melatonin upregulated APX and catalase, however, without substantially altering the production of superoxide and H_2_O_2_ under the influence of the oxidotoxin. Instead, melatonin promoted autophagy, which may be interpreted as a survival strategy to eliminate the oxidatively damaged organelles (Wang et al., 2015). In *Solanum lycopersicum*, cadmium toxicity was reduced by melatonin (Hasan et al., 2015). This was not restricted to the upregulation of antioxidant enzymes, but also included metal sequestration by phytochelatins and elimination of cadmium from the cytosol by transfer to the cell wall and to the vacuole.

Particular attention has been recently paid to actions of melatonin under conditions of biotic stress. This has been mainly studied in *Arabidopsis*, occasionally also in *Nicotiana*, using the pathovar tomato DC3000 of *Pseudomonas syringae*. First, melatonin was shown to upregulate pathogen-related, salicylic acid- and ethylene-dependent genes, effects that were suppressed in mutants defective in salicylic acid and ethylene signaling, such as *npr1*, *ein2*, and *mpk6* (Lee et al., 2014a). Next, *SNAT* knockouts did not only exhibit reduced levels of melatonin, but also of salicylic acid, along with a higher susceptibility to the pathogen (Lee et al., 2015). Infection with this pathogen caused rises in both melatonin and nitric oxide, while melatonin itself increased NO and salicylic acid-related genes, in conjunction with a reduced susceptibility (Shi et al., 2015a). Further studies revealed a relationship of melatonin to sugars that may also contribute to the thickness and resistance of cell walls (Zhao et al., 2015b). More detailed analyses (Qian et al., 2015) showed that melatonin enhanced the levels of various sugars, including glucose, fructose, sucrose, and melibiose, as well as that of glycerol, effects that were also elicited by the infection. Administration of fructose, glucose, sucrose, or glycerol increased pathogen resistance in wildtype plants, but not so in the salicylic acid-deficient NahG strain and in two NO-deficient mutants, *noa1* and *nia1nia2*. Moreover, the sugars and glycerol caused rises in NO but not in melatonin. In conclusion, melatonin seems to be involved in innate plant immunity as a factor that acts upstream of salicylic acid and NO, while rises in sugars and glycerol stimulate NO formation. With regard to the reduced resistance in *SNAT* knockouts, a full stimulation of immunity likely requires upregulation of melatonin synthesis.

## Photoreactions and Photoprotection

In the context of increased levels of melatonin and protection conveyed by this compound, most publications on exposure to high light intensities and especially UV have discussed this as just one of various forms of stress. However, there are several peculiarities that seem to exceed a more generalized stress response. First, the extent of UV-induced rises in melatonin is, in many cases, remarkably large. Alpine or Mediterranean species or varieties, which are exposed to high natural light and UV intensities, were shown to contain considerably higher melatonin levels than the same or related species from other habitats (Conti et al., 2002; Caniato et al., 2003; Hardeland et al., 2007b). Conti et al. (2002) published data of nine Alpine or Mediterranean plants with levels of 10–43 μg melatonin/g tissue. In fruits of an Egyptian *Fragaria* cultivar, high vis/UV light exposure was reported to increase melatonin (Badria, 2002). In leaves of the pontederiacean *E. crassipes*, melatonin levels were considerably higher (up to 300 ng/g fresh weight) in plants directly collected from a pond (maximal diurnal irradiation 10,000–15,000 μW/cm^2^) than those (ca. 3 ng/g) from laboratory conditions (400–450 μW/cm^2^; Tan et al., 2007). Presumably, this increase has included effects by UV, but the contribution of a temperature cycle in the natural environment cannot be ruled out. An aspect that speaks for a role of radiation and, perhaps, also for a photoprotective function of melatonin concerns the observed increase of melatonin in the course of the photophase, with a maximum around the transition to the scotophase. Notably, the diurnal change in melatonin concentration was accompanied by a corresponding rhythm in AFMK formation (Tan et al., 2007). As mentioned at the end of the section on metabolism, a contribution of indoleamine 2,3-dioxygenase to this rhythm cannot be excluded. However, melatonin is known to undergo several photocatalytically mediated reactions including dioxygenation (Behrmann et al., 1997; Hardeland et al., 2007b). Light-exposed extracts from phototroph organisms, such as the dinoflagellate *L. polyedrum* and the pheophycean *Pterygophora californica*, converted melatonin efficiently to AFMK, even in the presence of the hydroxyl radical scavenger DMSO (Hardeland et al., 1995; Behrmann et al., 1997). Among known light-induced reactants, melatonin is, in particular, oxidized by singlet oxygen to AFMK (de Almeida et al., 2003; Schaefer and Hardeland, 2009). The photoproduct AFMK is relatively inert against singlet oxygen and can, therefore, accumulate under these conditions. However, its secondary, deformylated metabolite, *N*^1^-acetyl-5-methoxykynuramine (AMK), which may be formed in plants enzymatically or by interactions with free radicals, is, among low molecular weight metabolites, one of the most efficient singlet oxygen quenchers detected, more potent than melatonin or histidine (Schaefer and Hardeland, 2009), and additionally an effective scavenger of various reactive oxygen and nitrogen species (Ressmeyer et al., 2003; Guenther et al., 2005; Hardeland et al., 2007a).

A presumed role of melatonin and, perhaps, its metabolites in photoprotection of plants would also be in line with the observed protection against chlorophyll degradation (Arnao and Hernández-Ruiz, 2009a; Tan et al., 2012; Wang et al., 2012, 2013), although this has certainly also to be seen under additional aspects of chlorophyll a/b binding protein (CAB) expression, delayed upregulation of pheophorbide a oxygenase (Wang et al., 2013) as well as stress- and senescence-related signaling.

However, the assumed photoprotection of plants should not be generalized. In *Glycyrrhiza uralensis*, exposure to visible or UV-B light caused rises in melatonin, but mainly in the roots (Afreen et al., 2006), a response that should rather be interpreted as a stress response. Moreover, high light intensities do not generally favor an elevation of melatonin. In *Oryza sativa*, a heat-induced increase was antagonized by light (Byeon and Back, 2014).

A further aspect, which merits more future attention, concerns the concentration dependence of protection. Very high melatonin levels in leaves, as sometimes observed in strongly UV-exposed plants (Conti et al., 2002; Caniato et al., 2003), may allow nonenzymatic elimination of free radicals and singlet oxygen at substantial rates, in addition to the anyway effective antioxidative protection system based on ascorbate, glutathione, APX, glutathione peroxidase (GPX) and other compounds. Moreover, two properties of melatonin should be considered, namely, a nonadditive synergistic interaction of melatonin and other antioxidants, notably, ascorbate and glutathione (Tan et al., 2003) and melatonin’s scavenger cascade, which allows elimination of up to 10 free radicals per melatonin molecule by formation of consecutively formed metabolites with scavenging properties (Rosen et al., 2006). The highly divergent levels of melatonin found in different plant species may allow, in some of them, a substantial contribution to nonenzymatic protection, whereas, in other species containing this compound in much lower concentrations, actions are restricted to signaling mechanisms. The background of this divergence may be sought in unfavorable physiological effects of elevated melatonin in certain species. Such a case was recently reported. In *Zea mays*, low doses of melatonin favored photosynthesis and nocturnal starch catabolism, whereas high doses caused the opposite, downregulated sucrose transporter expression and inhibited seedling growth (Zhao et al., 2015a). In conclusion, physiologically relevant differences in melatonin tolerance may exist between plant species.

## Conclusion

During the last years, considerable progress has been made in melatonin research in plants. This does not only concern the extension of knowledge on its presence in an increasing number of species and taxa, but also the identification of metabolic pathways and a remarkable amount of new information on the involvement in physiological functions. Nevertheless, there is still a strong demand for clarification of several fundamental points.

One of these is related to the enormous differences in the melatonin content of different species and organs. As already outlined in the Introduction, signaling mechanisms based on high-affinity binding sites for melatonin cannot be expected to work at strongly elevated concentrations that would permanently saturate a binding protein. However, this aspect of divergent concentrations requires a distinction between metabolically active and dormant tissues. In the case of extremely high melatonin levels in leaves, signaling mechanisms known from low-melatonin species may not be functional. Alternately, one would have to assume either different mechanisms based on low-affinity instead of high-affinity binding sites or a sequestration of melatonin in areas of limited metabolic activity, such as the vacuole or the apoplast. The necessity of determining the distribution of melatonin within the tissue (Hardeland, 1997) might resolve some concentration-related discrepancies that appear rather enigmatic to date. An additional possibility of removing melatonin from the cytosol might consist in its uptake into oil bodies, which has been shown to occur in sunflower seedlings (Mukherjee et al., 2014). Moreover, this could be of particular importance in oily seeds. High melatonin levels in seeds of various plants are well documented (Manchester et al., 2000). A more or less dry, dormant seed is practically devoid of enzymatic antioxidative protection and has, therefore, to rely on low molecular-weight antioxidants (Balzer and Hardeland, 1996; Hardeland et al., 2007b), among which melatonin has a number of advantages. Moreover, melatonin might contribute to the maintenance of the dormant state and to the survival in dormancy (Balzer and Hardeland, 1996; Hardeland et al., 2007b), at least by reducing oxidative damage, which limits the germination potential of seeds. In fact, the preservation of seed viability by applying high concentrations of exogenous melatonin has been recently demonstrated in *Arabidopsis* (Hernández et al., 2015). In rehydrated seeds, melatonin may promote seed germination, especially under unfavorable conditions. This view is supported by recent findings in seeds of *Cucumis sativus* under high salinity (Zhang et al., 2014). Support of germination was also reported for the negatively photoblastic seeds of *Phacelia tanacetifolia*, but interpreted, in this case, as a mimicking of darkness by melatonin (Tiryaki and Keles, 2012).

Another aspect that awaits further clarification concerns the role of isoenzymes in the melatonin biosynthetic pathway, as outlined in the section on metabolism. More specifically, the contribution of different isoenzymes to the highly divergent melatonin levels detected in different species should be elucidated.

The field in which progress is most urgently required is that of signaling pathways. Although the influence of melatonin on gene expression otherwise known to be controlled by phytohormones has been amply documented by transcriptomic and proteomic analyses, primary signaling mechanisms by melatonin have remained entirely unexplored. With regard to the cytoskeletal effects, binding of melatonin to calmodulin may occur, as known from animals, but has not been directly demonstrated. However, high-affinity binding sites of melatonin and their properties are still unknown in plants. In the absence of their identification, no information can be obtained on receptor-mediated signal transduction and second messengers involved in the modulation of actions of established phytohormones and gene regulation. Progress in this field may be critical to the possible classification of melatonin as a phytohormone.

## Author Contributions

The author confirms being the sole contributor of this work and approved it for publication.

## Conflict of Interest Statement

The authors declare that the research was conducted in the absence of any commercial or financial relationships that could be construed as a potential conflict of interest.
